# Shaping the Infant Microbiome With Non-digestible Carbohydrates

**DOI:** 10.3389/fmicb.2019.00343

**Published:** 2019-02-25

**Authors:** Stella Verkhnyatskaya, Michela Ferrari, Paul de Vos, Marthe T. C. Walvoort

**Affiliations:** ^1^Stratingh Institute for Chemistry, Faculty of Science and Engineering, University of Groningen, Groningen, Netherlands; ^2^University Medical Center Groningen, Groningen, Netherlands

**Keywords:** infant, microbiome, non-digestible carbohydrates, exopolysaccharides, transglycosylation

## Abstract

Natural polysaccharides with health benefits are characterized by a large structural diversity and differ in building blocks, linkages, and lengths. They contribute to human health by functioning as anti-adhesives preventing pathogen adhesion, stimulate immune maturation and gut barrier function, and serve as fermentable substrates for gut bacteria. Examples of such beneficial carbohydrates include the human milk oligosaccharides (HMOs). Also, specific non-digestible carbohydrates (NDCs), such as galacto-oligosaccharides (GOS) and fructo-oligosaccharides (FOS) are being produced with this purpose in mind, and are currently added to infant formula to stimulate the healthy development of the newborn. They mimic some functions of HMO, but not all. Therefore, many research efforts focus on identification and production of novel types of NDCs. In this review, we give an overview of the few NDCs currently available [GOS, FOS, polydextrose (PDX)], and outline the potential of alternative oligosaccharides, such as pectins, (arabino)xylo-oligosaccharides, and microbial exopolysaccharides (EPS). Moreover, state-of-the-art techniques to generate novel types of dietary glycans, including sialylated GOS (Sia-GOS) and galactosylated chitin, are presented as a way to obtain novel prebiotic NDCs that help shaping the infant microbiome.

## Introduction

Humans live in symbiosis with trillions of bacteria, and most of them are symbionts and beneficial to the host (Sender et al., 2016). Disturbance in our microbiota can contribute to the development of many diseases (Wang et al., 2017). Bacteria are mainly present in the areas that are more exposed to the surrounding environment such as the skin, vaginal and oral mucosa, and the GIT. The gut microbiota has been extensively studied due to its impact on the establishment of immunity (Martin et al., 2010) and prevention of chronic inflammation (Belkaid and Hand, 2014). While the fetal GIT was considered sterile for many years, emerging evidence suggests that colonization of the GIT starts already at the prenatal stage with neonatal colonization by *Enterobacter*, *Escherichia*, *Shigella*, and *Staphylococcus* species, as detected in the umbilical cord, placenta, and amniotic fluid (Carmen Collado et al., 2016). After birth, the newborn gut is rapidly colonized by different bacterial strains with the first colonizers being facultative aerobes such as *Escherichia* and *Enterococcus*, whose oxygen consumption allows colonization of anaerobic bacteria, with the most abundant being *Bifidobacterium* (Houghteling and Walker, 2015). Many early-life factors have an impact on the composition of the infant gut microbiota, including the mode of delivery, the infant feeding pattern, diet composition, and the use of antibiotics, but also the health of the mother during pregnancy (Gonzalez-Perez et al., 2016).

The early colonization process is crucial for a healthy microbiome and prevents disease later in life. Gut microbiota are essential for digestion of food, but also to function as a barrier against pathogens, and for the development of immune tolerance to innocuous antigens and microorganisms (Yang et al., 2016). Imbalances in the intestinal microbiome composition can result in bacterial overgrowth or lower species diversity, making the host more susceptible to pathogenic infections (Lozupone et al., 2012). Furthermore, microbial dysbiosis may lead to autoimmune and allergic diseases. The healthy infant intestinal microbiome has a low microbial diversity, with *Bifidobacterium*, *Bacteroidetes*, *Firmicutes*, and *Proteobacteria* being most abundant. Feeding has a major influence on the microbiota composition, as breast-fed infants have higher *Bifidobacterium* and *Enterobacteria* numbers and a lower diversity in comparison to formula-fed infants (Milani et al., 2017).

There is a growing understanding of the mechanisms by which a balanced microbiome contributes to health. For instance, many genera such as *Eubacterium* and *Bacteroides* are involved in the production of vitamin K (Rossi et al., 2011), an essential cofactor promoting the γ-carboxylation of glutamate residues involved in blood clotting (Gröber et al., 2014). *Bifidobacterium* species are able to produce folate, a vitamin involved in DNA synthesis and repair with an undisputed importance in neurological development (Crider et al., 2012), with the best producing strains being *Bifidobacterium adolescentis* and *Bifidobacterium pseudocatenulatum* (Rossi et al., 2011). Lactobacilli carry the *rib* operon, which is implicated in the *de novo* synthesis of riboflavin, which is important in developmental processes and in the hemopoietic system (Thakur et al., 2016). Moreover, gut microbiota are responsible for the production of SCFAs, such as acetate, propionate, and butyrate. Acetate is the most abundant, and it is used by many gut commensals to produce propionate and butyrate in a growth-promoting cross-feeding process. SCFAs are important for the reduction of the intestinal pH and the consequent inhibition of pathogen’s adhesion. Moreover, butyrate is the preferred energy source for colon epithelial cells, where it contributes to the maintenance of the gut intestinal barrier, exerts immunomodulatory and anti-inflammatory effects (Stilling et al., 2016; Zhang et al., 2018), also through epigenetic mechanisms (Furusawa et al., 2013; Paparo et al., 2014), and may even prevent colorectal cancer (Wu et al., 2018).

A healthy infant microbiome is normally created under the guidance of molecules in human milk. This is mainly accomplished by HMOs, which serve as feed for specific bacterial species. HMOs are a family of >200 structurally different molecules that vary in quantity and composition from mother to mother, and over the course of lactation. However, some general trends in HMO composition are present (Table 1). HMOs are composed of a linear or branched backbone containing galactose (Gal), *N*-acetylglucosamine (GlcNAc), and glucose (Glc), which can be decorated with fucose (Fuc) and sialic acid (Sia) residues, and this decoration pattern depends on the mother’s secretory status (Bode, 2012). Only members of *Bifidobacterium* and *Bacteroides* were shown to metabolize HMOs (Marcobal et al., 2010). Especially *Bifidobacterium bifidum* and *Bifidobacterium infantis* are efficient utilizers of HMOs, whereas they are moderately digested by *Bifidobacterium breve* and *Bifidobacterium longum*. Interestingly, *Bifidobacterium animalis* and *B. adolescentis* are incapable of degrading HMOs (LoCascio et al., 2009; Sela and Mills, 2010). To ensure a high number in the gut, bifidobacteria have been observed to create a cross-feeding niche, as the extracellular fermentation of HMOs by *B. bifidum* is associated with a cooperative effect for *B. infantis*, which is able to import the released sugars and digest them intracellularly (Garrido et al., 2012; Thomson et al., 2018).

**Table 1:** Overview of oligosaccharide structures discussed herein.

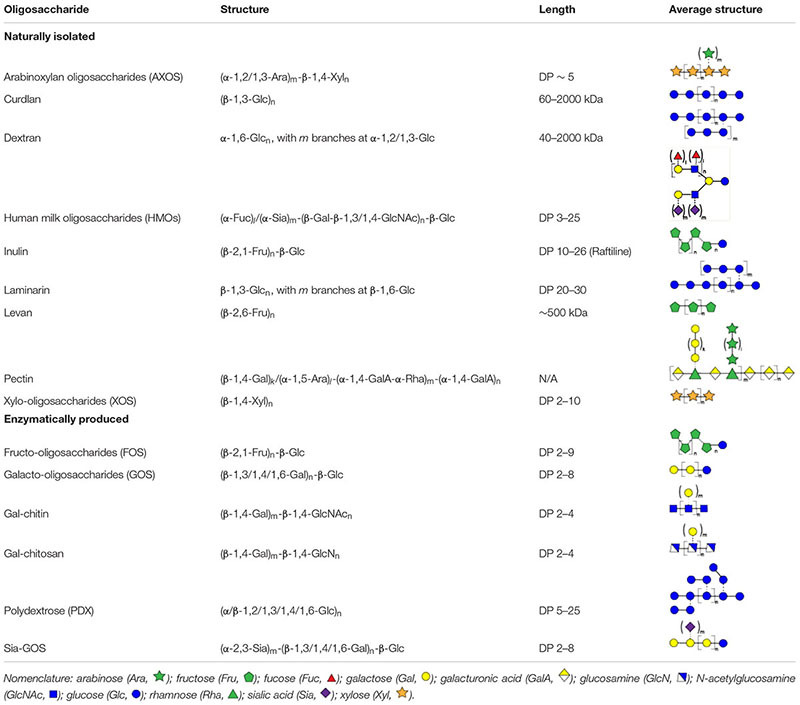

For infants where human milk is not an option, infant formula supplemented with NDCs that should mimic prebiotic functions of HMOs have been created (Vandenplas et al., 2015). A prebiotic is defined as “a substrate that is selectively utilized by host microorganisms conferring a health benefit” (Gibson et al., 2017). HMOs fulfill these criteria, as they are not digested in the upper part of the GIT of infants (Engfer et al., 2000), while they serve as preferred food source for beneficial bacteria. Next to HMOs, other NDCs or dietary fibers have been shown to be major drivers of gut microbiome composition and function, and might be added to infant formula for this purpose (Benitez-Paez et al., 2016). Interestingly, the currently applied molecules do not mimic all the functions of the >200 HMOs found in human milk, so novel oligosaccharides are needed to fill this void. This review aims to inspire the selection of future NDCs that can be added to infant formula by reviewing beneficial glycans that show great promise as modulators of the microbiome, with a focus on their interaction with bifidobacteria and lactobacilli, since most is known about these genera. Moreover, state-of-the-art techniques to generate novel types of dietary glycans are presented.

## NDCs Currently Added to Infant Formula

To mimic the beneficial effects of HMOs, two alternative oligosaccharides are routinely added to infant formula: GOS and FOS (Table 1). GOS are produced by enzymatic transglycosylation from lactose (vide infra), providing a mixture of differently linked oligosaccharides with a DP from 2 to 8. The Gal units are linked through β-galactosidic linkages, which are resistant to GIT enzymes until they reach the colon where they are fermented by bacteria. In general, GOS stimulate the growth of bifidobacteria (Absmanner et al., 2010), and especially the numbers of *B. adolescentis* are impacted (Sierra et al., 2015). FOS are generally produced by enzymatic digestion from naturally isolated inulin, yielding oligosaccharides with DP from 2 to 9, and bifidobacteria readily grow when FOS are used as a sole carbon source (Macfarlane et al., 2008). When mixtures of GOS/FOS in a 9/1 ratio are used, the ratio of different *Bifidobacterium* species was similar to breast-fed infants (Haarman and Knol, 2005). This GOS/FOS mixture was also demonstrated to be the best growth substrate for *Bifidobacteria* and *Lactobacilli*, while inulin and PDX led to poor growth (Vernazza et al., 2006). PDX is a synthetic polymer of randomly connected Glc units with an average DP of 12 and all possible glucosidic linkages: α- or β- and 1→2, 1→3, 1→4, and predominantly 1→6 (Ramiro do Carmo et al., 2016). When PDX was used in combination with GOS in a 1:1 ratio, the increase in *Bifidobacterium* species, specifically *B. infantis*, *B. longum*, and *B. catenulatum*, was similar to the breast-fed microbiota, where *B. infantis*, *B. longum*, and *B. breve* are predominant (Scalabrin et al., 2012). Interestingly, this GOS/PDX mixture was also identified in a commercial brand of infant formula (Nijman et al., 2018). Next to prebiotic properties, GOS, FOS, and mixtures of both components were also shown to have immunomodulatory properties, which have recently been reviewed (Macfarlane et al., 2008; Ackerman et al., 2017, Akkerman et al., 2018).

## Alternative NDCs Isolated From Natural Sources

Polysaccharides with prebiotic potential have mostly been extracted from the cell wall of higher plants including cereals and grains, fruits, and vegetables, seaweeds, and microalgae (de Jesus Raposo et al., 2016). In this section, we focus on the naturally isolated polysaccharides POS and AXOS that have already been investigated for their prebiotic effect and might serve as alternative for HMOs.

Pectins have received widespread attention for their potential as prebiotics. They are composed of a backbone of galacturonic acids, which are hypothesized to mimic the Sia residues in HMOs (Table 1). Pectins are heteropolysaccharides and are available from citrus peels, apple pomace, sugar beet pulp, and potato pulp. The hydrolysis of pectins yields POS, which are composed of galacturonic acid, galactose, rhamnose, arabinose, and xylose building blocks. Moreover, POS can be methylated or esterified on the galacturonic acid residues, and the degree of methylation, esterification, and the ratios of monosaccharides depends on the source of pectin and the type of extraction method used. In light of this structural diversity, studies with POS become more reliable and reproducible when the exact molecular structure is described. POS has a demonstrated prebiotic effect, promoting the growth of *Bifidobacteria* and *Lactobacilli*. Interestingly, especially neutral POS, such as galactan, GOS, arabinan, and arabino-oligosaccharides, enhance the growth of *Bifidobacteria* to a similar extent as inulin (Onumpai et al., 2011; Di et al., 2017). A similar increase in bifidobacteria numbers was observed for an arabinose-rich mixture of SB-POS, while lactobacilli were selectively enhanced using lemon peel waste-derived POS, which was high in galacturonic acids, and the number of bacterial members of *Faecalibacterium prausnitzii* group and *Roseburia intestinalis* (both of the phylum Firmicutes) increased with all types of pectins (Gomez et al., 2016). In contrast, a commercial source of SB-POS, which was shown to contain a high galacturonic acid content, had little effect on numbers of bifidobacteria, highlighting the importance of the pectin composition (Leijdekkers et al., 2014). Infant formula with pectins has been studied in human infant trials, but there was no effect of the acidic oligosaccharides on bifidobacteria and lactobacilli (Fanaro et al., 2005).

Xylo-oligosaccharides (XOS, Table 1) are present in fruits, vegetables, bamboo, honey, and milk, and can be produced on an industrial scale by enzymatic degradation of xylan-rich materials (Aachary and Prapulla, 2011). XOS is readily fermented by commensal bacteria, and can in humans increase the population of fecal bifidobacteria and SCFA production (Lecerf et al., 2012). AXOS (Table 1) are prepared by degradation of arabinoxylan, which is the major non-cellulose polysaccharide in cereals and plants. In a fermentation study, it was shown that *B. longum* B24 could liberate the arabinose units from AXOS without degrading the xylan backbone, while *B. longum* B18 was able to metabolize XOS up to DP4 (Riviere et al., 2018). *B. adolescentis* B72 degraded various types of FOS, partially degraded inulin, and metabolized XOS longer than DP4. The authors suggested that the strain-specific mechanisms to utilize different glycans lead to a cooperative effect and simultaneous striving of different bacterial strains. A similar cross-feeding effect was observed between *B. longum* NCC2705 and *Eubacterium rectale* ATCC 33656 when grown on AXOS (Riviere et al., 2015). *B. longum* is able to release arabinose and produce acetate, whereas *E. rectale* uses acetate to produce butyrate. When co-cultured on AXOS, the consumption of arabinose by *B. longum* and concomitant release of acetate allowed *E. rectale* to produce butyrate, resulting in a simultaneous prebiotic and butyrogenic effect (Riviere et al., 2016). Other examples of such a commensal cross-feeding relationship with bifidobacteria have been reported, including *Faecalibacterium* (De Vuyst and Leroy, 2011; Moens et al., 2016). Negatively charged XOS structures, containing glucuronic acid units, have also been isolated from hardwood (Rivas et al., 2017), and may be promising candidates for novel charged prebiotic NDCs (vide infra).

## Potential of Exopolysaccharides as Novel NDCs

Exopolysaccharides produced by Gram-positive bacteria currently attract a great deal of attention because of their wide range of beneficial properties (Ryan et al., 2015). Regularly new EPS structures are identified that have a specific health effect, and especially the immune-modulating properties are often investigated (Castro-Bravo et al., 2018). From recent reviews on the characterized EPS structures of *Lactobacillus* and *Bifidobacterium*, their great structural diversity is immediately apparent (Hidalgo-Cantabrana et al., 2014; Castro-Bravo et al., 2018; Oleksy and Klewicka, 2018). They are broadly divided into HoPS, which are composed of a single sugar building block, and HePS, which display a repeating fragment of two to eight different sugar units.

Most HoPS are found to be susceptible to fermentation by commensal bacteria (Salazar et al., 2016), which is presumably directly linked to their relatively simple molecular structure, albeit that they can be very large in size. For instance, the prebiotic effect of β-fructans was investigated with two levan-type EPS isolated from *Lactobacillus sanfranciscensis*, and compared with levan (fructan with β-2,6 linkages, Table 1), inulin (fructan with β-2,1 linkages), and FOS (Dal Bello et al., 2001). An enrichment of *Bifidobacterium* species in human fecal samples in a large bowel model medium was observed with the EPS and inulin as added carbon source, while levan and FOS had no effect. This may reflect the importance of both the length of the carbohydrate, and the fructose linkage type in the isolated EPS, which may be different from commercial levan. The capability of *Bifidobacterium* species to directly metabolize the *L. sanfranciscensis* EPS was further demonstrated in a fermentation study (Korakli et al., 2002). β-Glucans, including curdlan (linear β-1,3-Glc, Table 1) and laminarin (β-1,3/1,6-Glc, Table 1), are also readily fermented by bifidobacteria. Especially the *B. infantis* population benefitted from β-glucan digestion, and concomitant increased production of propionate and butyrate was observed (Zhao and Cheung, 2011).

In contrast, there is a lack of data on the digestibility of HePS by commensal bacteria, presumably due to their complex structures and generally low isolated yields. Both bifidobacteria and lactobacilli display structurally diverse HePS, which may contain galacto-pyranose and -furanose, rhamnose, mannose, and 6-deoxy-talose, among others (Hidalgo-Cantabrana et al., 2014). In a fecal slurry fermentation experiment, the uncharacterized EPS from different *B. animalis*, *B. pseudocatenulatum*, and *B. longum* species isolated from humans were investigated for their prebiotic effect (Salazar et al., 2008). Although there were high inter-individual variations, the data indicated an EPS-related enrichment of *Bifidobacterium* species, similar to the result obtained with inulin. *Bacteroides fragilis* DSMZ 2151 was also found to digest (uncharacterized) HePS from *B. longum* E44 and *B. animalis* subsp. *lactis* R1, with concomitant increase in propionate and acetate production (Rios-Covian et al., 2016). Although there is no data on fermentation yet, an interesting link between acidic phosphate groups in HePS structures and immune responses was found (Kitazawa et al., 1998). *Lactobacillus delbrueckii* subsp. *bulgaricus* OLL-1073-R1 produces two different EPS: acidic phosphate-containing (APS) and NPS, both composed of Glc and Gal residues (ratio 3:2). Interestingly, only the APS was a strong inducer of proliferation and activity of macrophages. When the APS was fractionated in two different EPS based on size, the B-cell mitogenic activity was observed only with high-molecular weight polysaccharide (H-APS). The impact of the acidic phosphate was substantiated by chemical dephosphorylation, which resulted in a reduction of the stimulatory effect (Kitazawa et al., 1998). Interestingly, when unrelated dextran (α-Glc HoPS from *Leuconostoc mesenteroides*, Table 1) was chemically phosphorylated, the proliferation of lymphocytes was directly proportional to the phosphate content (Sato et al., 2004). Unfortunately, there is no information available on the fermentability of these charged EPS, which could shed a light on their prebiotic potential. Overall, the structural complexity of especially the HePS yields large promise for prebiotic potential, which warrants extra dedication to unraveling the molecular structure of prebiotic HePS to gain more insight in the structure–function relation.

## Development of Novel NDCs

With the increasing interest and appreciation of the impact of dietary glycans on healthy microbiome development and overall human health, there is a tremendous surge in methods to produce existing and novel glycans. Chemical synthesis has the potential to generate well-defined carbohydrate structures, but reliable methods are not generally available, and especially not on the scale that would allow for biological evaluation. Enzymatic synthesis is more amenable to larger scale carbohydrate production, but also has its challenges. GTs have successfully been used in the synthesis of HMO structures *in vitro* (Chen et al., 2015; Yu et al., 2017a), but their application is hampered by the use of expensive nucleotide-activated sugars, and multi-enzyme substrate recycling systems are needed to prevent metabolites from inhibiting enzyme activity (Qin et al., 2016). Using bacterial cells as production factories however, major advancements in HMO production have been made and have resulted in FDA approval and commercialization of the major HMO 2′-fucosyllactose. Different methods are now available in *Saccharomyces cerevisiae* (Yu et al., 2018) and *Escherichia coli* (Chin et al., 2017), and other HMO structures are expected to be produced in this way in the near future (Sprenger et al., 2017). Alternative methods rely on the use of GHs, which are able to perform a transglycosylation reaction next to glycosidic bond hydrolysis (Danby and Withers, 2016; Manas et al., 2018). In this way, well-known prebiotic fibers such as GOS are industrially produced by making use of β-galactosidase enzymes (Torres et al., 2010), and also FOS can be synthesized in this way (Karboune et al., 2018). This approach can also be used to decorate existing glycans with other sugars, and the generation of galactosylated, fucosylated (Zeuner et al., 2018), and sialylated glycans as HMO mimics have recently been reviewed (Zeuner et al., 2014). A variety of glycan acceptors, ranging from monosaccharides and lactose to Tn antigens (e.g., *N*-acetylgalactosamine-threonine conjugates), GOS, and HMOs have been described. This strategy has the potential to rapidly yield novel dietary glycans that display complex sugar building blocks (e.g., Sia, Fuc) that were previously difficult to obtain.

A successful example of this strategy is the production and biological evaluation of sialylated GOS (Sia-GOS, Table 1). Using a transsialidase from *Trypanosoma cruzi* and bovine κ-casein-derived GMP as the source of Sia, commercial GOS was decorated with α-2,3-Sia residues to create mono-Sia-GOS (Wilbrink et al., 2015). These novel glycans were subsequently tested in a rat model of NEC, an intestinal disorder mainly observed in preterm infants, for which sialylated HMOs were found to protect (Jantscher-Krenn et al., 2012; Yu et al., 2017b). Interestingly, Sia-GOS significantly reduced the pathology score of NEC, with pooled HMO still being superior in terms of protection, while regular GOS supplementation and formula-feeding both resulted in the worst pathology scores (Autran et al., 2016). In separate fermentation studies, with a Sia-GOS batch produced by a GT-catalyzed sialylation, it was revealed that *B. infantis* ATCC 15697 was able to digest Sia-GOS, whereas *B. adolescentis* ATCC 15703 could not, highlighting the species-specific ability to metabolize HMOs and HMO mimics (Wang et al., 2015).

Using a similar strategy, chitin and chitosan (deacetylated at the amine) oligosaccharides were decorated with β-Gal residues (Black et al., 2014). The transglycosylation was performed with β-galactosidase from *Lactobacillus plantarum* with lactose as the Gal source, and different chitin and chitosan acceptors were decorated with one to three residues in a β-1,4 linkage (Table 1). Especially the β-Gal-chitosan and GOS oligosaccharides were found to prevent enterotoxigenic *E. coli* K88 from adhering to porcine erythrocytes, in contrast to alpha-linked GOS and α-Gal-chitosan (Yan et al., 2017; Yan and Ganzle, 2018). It will be interesting to perform digestion studies of these novel β-Gal-chitosan glycans by bacteria to investigate their prebiotic effect.

## Concluding Remarks

It is clear that the creation of a healthy infant microbiome is a delicate interplay of a variety of commensal bacteria, which can be beneficially influenced by oligosaccharides. Because the composition of the infant’s microbiome can have a profound effect on adult life, there is a great potential for the addition of carbohydrates that mimic HMO functions. Promising better candidates that may substitute or be added to currently applied NDCs are the HePS, which have the potential to specifically enhance certain species. Also, as structural mimics of HMOs, fucosylated and sialylated oligosaccharides are expected to be applied in the near future. In the end, more knowledge of the presence of the biosynthetic machinery necessary to utilize specific oligosaccharides will pave the way for the development of novel NDCs with prebiotic effects.

## Author Contributions

SV, MF, and MW contributed to the organization and structure of the review. All authors contributed to the writing and critical evaluation of the final version.

## Conflict of Interest Statement

The authors declare that the research was conducted in the absence of any commercial or financial relationships that could be construed as a potential conflict of interest.

## Abbreviations

APS: acidic polysaccharides
AXOS: (arabino-)xylo-oligosaccharides
DP: degree of polymerization
EPS: exopolysaccharides
FOS: fructo-oligosaccharides
GHs: glycosyl hydrolases
GIT: gastrointestinal tract
GMP: glycomacropeptide
GOS: galacto-oligosaccharides
GTs: glycosyl transferases
H-APS: high-molecular weight acidic polysaccharides
HePS: heteropolysaccharides
HMOs: human milk oligosaccharides
HoPS: homopolysaccharides
NDCs: non-digestible carbohydrates
NEC: necrotizing enterocolitis
NPS: neutral polysaccharides
PDX: polydextrose
POS: pectin oligosaccharides
SB-POS: sugar beet pulp pectin oligosaccharides
SCFAs: short chain fatty acids.
